# Prevalence and pattern of self-medication in Karachi: A community survey

**DOI:** 10.12669/pjms.315.8216

**Published:** 2015

**Authors:** M. Iqbal Afridi, Ghulam Rasool, Rabia Tabassum, Marriam Shaheen, Siddiqullah, M. Shujauddin

**Affiliations:** 1 Prof. M. Iqbal Afridi, FCPS, FRCP. Head, Dept. of Psychiatry & Behavioural Sciences, Head, Dept. of Neurology, Secretary, Faculty of Psychiatry, College of Physicians & Surgeons, Pakistan. Jinnah Post Graduate Medical Centre (JPMC), Karachi, Pakistan; 2 Dr. Ghulam Rasool, MBBS. Postgraduate FCPS-II Trainee, Dept. of Psychiatry & Behavioural Sciences, Jinnah Post Graduate Medical Centre (JPMC), Karachi, Pakistan; 3 Dr. Rabia Tabassum, MBBS; 4 Dr. Marriam Shaheen, MBBS. Dow University of Health Sciences, Karachi, Pakistan; 5 Dr. Siddiqullah, MBBS. Dow University of Health Sciences, Karachi, Pakistan; 6 Dr. M. Shujauddin, MBBS. Dow University of Health Sciences, Karachi, Pakistan

**Keywords:** Self-medication, Over-the-counter medicines, Non-prescribed medicines

## Abstract

**Objective:**

To study the prevalence and pattern of self-medication among adult males and females in Karachi, Pakistan.

**Methods:**

This cross-sectional community- based survey was carried out at five randomly selected towns of Karachi (Defence, Gulshan-e-Iqbal, North Nazimabad, Malir, Orangi town) over a period of 3 months (October, November & December 2012). A sample size of 500 adult cases (250 males & 250 females), with systemic random selection from different towns of Karachi were inducted in this study. The city was divided in 5 zones and one town from each zone was selected by systemic randomization. First available male and female from each randomly selected house were included in the study. After consent and confidentiality assurance they were interviewed on semi-structured Performa designed for this purpose. Results were analyzed and tabulated through SPSS v14.0.

**Result:**

The prevalence of self-medication in males and females in Karachi is found to be 84.8% (males 88.4% and females 81.2%). The most frequent symptoms for which self-medication used were headache (32.7%), fever (23.3%) and the medicines used were painkillers (28.8%), fever reducer medicines (19.8%). The most common reason 33.3% was previous experience with similar symptom.

**Conclusion:**

Self-medication is highly prevalent (84.8%) in Karachi. It was frequently used for headache followed by fever. Predominantly painkillers, fever reducer and cough syrups were used in the form of tablets and syrups. Main source of medicines for males were friends and for females were relatives.

## INTRODUCTION

Self-medication can be defined as obtaining and consuming drug(s) without the advice of a physician either for diagnosis, prescription or surveillance of treatment.^1^

In studies it is reported that self-medication is widely practiced in developing^2^, as well as in developed countries.^3^,^4^ The use of Over-the-Counter (OTC) drugs has been studied in many different populations and the results demonstrate that about 25-75% of the population consume OTC medications,^5^,^6^ it has been noticed when people get sick they prefer to consult a chemist or neighbor or search internet or magazines rather to consult a physician and this raises concerns of incorrect self-diagnosis, drug interaction, and use other than for the original indication.^7^

Experts are worried that OTC drugs allow people to ignore serious symptoms until it is too late. Combining medications is another problem doctors encounter with users of OTC. In the interest of profit, the OTC industry continues to propagate the idea that OTCs are completely safe; however more and more doctors are speaking out against their widespread usage.^8^ Although there are rules for OTC but the gravity of the problem can be serious in developing countries where implementation is generally lacking.

The prevalence rates of self-medication are high all over the world; up to 68% in European countries^4^ while much higher in the developing countries.^2^ It is as high as 98% in Palestine.^9^ In a drug utilization study in USA, it was found that medications that are contraindicated in pregnancy were used at unexpectedly high rate as OTC drugs in obstetric population.^10^ Very few studies in Pakistan that too were representing a specific group of people regarding self-medication have been conducted which have also confirmed high rates of prevalence of around 51%.^11^ In rural areas, this rate is much higher because of the lack of healthcare infrastructure. Neither medicines nor doctors are easily available to 70% population of Pakistan.^12^ It is also alarming that the prevalence rates are on the rise despite efforts to limit this problem.^13^

There is a trend towards use of ayurvedic and homeopathic drugs for chronic illnesses like joint pains, acid peptic disease, bronchial asthma, obesity, impotence, baldness and female infertility. Moreover, herbs and homeopathic drugs were considered safe and devoid of adverse effects, but the risk of possible drug interactions are always there with their use.^14^

In Pakistan, almost every pharmacy sells drugs without a prescription; a phenomenon seen in many developing countries. Consequently, antibiotics and potentially habit-forming medicines especially benzodiazepines are easily available to the common man. This together with poor awareness leaves the layman uninformed about the potentially lethal effects of some of these drugs. The lack of a good primary health care system coupled with cost issues compel the general public to approach other avenues instead of a doctor’s to seek help for a problem. Despite this, there is paucity of literature regarding self-medication in Pakistan and there is dire need to address this problem.^15^

Keeping in mind the above facts, this study was designed to understand the phenomena of self-medication in local contest in terms of frequency, pattern and gender.

## METHODS

This community-based cross-sectional study was initially conducted as a pilot study on 20 cases in order to know the limitations of the Performa and it’s applicability in local socio-cultural setup. Some of the questions and wordings were modified based on the understanding and comprehension of the general public. A sample of 500 people, which comprised of 250 males & 250 females with age> 18 years, were taken from Karachi, over a period of 3 months (October, November & December 2012). The city comprises 5 zones. One town from each zone was selected by systemic randomization in order to represent various socio-economic groups and in this regard the selected towns were Defence, Gulshan town, Malir town, North Nazimabad town and Orangi town. One hundred equal samples were taken from each town by random sampling method. First available male and female were included in the study from each house. A semi-structured Performa based on simple questionnaire was designed and administered on each consenting individuals. Ethical issues (consent & confidentiality) were taken into account with approval of IRB, Jinnah Postgraduate Medical Centre (JPMC), Karachi. There were some hindrances in the form of socio-culturally *pardah* observing ladies. In those cases they were interviewed by female members of the team. Results were analyzed and tabulated using SPSS v14.0

### Exclusion Criteria

Person below 18 years, not giving consent, having communication problem, unable to understand because of severe illness, unconsciousness or mental retardation.

## RESULTS

Five hundred participants (250 males, 250 females) were randomly selected. Their mean age ±SD was 33.49 ±12.4 years (males 33.53 years, females 33.45 years) [Table-I].

**Table-I:** Socio-demographic data (n=500)

Variables	Males %	Females %	Total %
Age Mean±SD	33.45±12.97	33.53±11.8	33.49±12.4
Marital Status			
Married	52.4	64.8	58.6
Single	37.6	24.4	31.0
Others	10	10.8	10.4
Mother Tongue			
Urdu	58.8	58.0	58.4
Punjabi	18.0	18.0	18.0
Others	23.2	24	23.6
Education			
Inter	23.2	16.4	19.8
Graduate	19.2	26.8	23.0
Others	57.6	56.8	57.2
Occupation			
Business	21.6	1.6	11.6
Skilled Prof.	16.4	12.4	14.4
Student	12.4	14.8	13.6
House hold	1.2	65.2	33.2
Others	48.4	6	27.2

The reported self-medication was 84.8% population (males 88.4%, females 81.2%). Interestingly it was revealed that only 28.2% could understand what is meant by self-medication. The medicines used included 28.8% painkillers (31.5% males, 25.6% females), 19.8%fever reducer medicines (22.0% males, 17.9% females) [Table-II].

**Table-II:** Medicine used (n=500).

Types	Male %	Female %	Total %
Painkillers	31.5	25.6	28.6
Fever reducer drugs	22.0	17.9	20
Anti-allergy	6.0	5.6	5.8
Cough syrups	16.4	11.7	14.0
Antidepressants	0.4	0.1	0.2
Anti-diarrhea	4.9	5.5	5.2
Antibiotics	1.1	1.2	1.2
Multivitamins	3.7	5.3	4.5
Others	1.6	2.5	2.1
Supplements	0.5	2.1	1.3
Sleeping pills	0.7	0.9	0.8
Birth control pills		0.7	0.7
Weight reducing pills	0.5	1.8	1.2
Sexual activity enhancing	0.7	0.1	0.4
Homeopathic medicines	4.8	6.5	5.6
Herbal medicines	2.5	4.3	3.4
Home remedies	2.8	8.3	5.6

It was revealed that 33.3% were using it in the light of previous experience with same medication. The most common route of administration was oral in the form of tablets and syrups. Males predominately use it after meals while females at bed time. About 31.1% males while 51% females were using self-medication for years. For further details refer [Table-III].

**Table-III:** Finding from the questionnaire regarding self-medication.

Variables	Male %	Female %	Total (%)
*Form of Self-Medication*			
Tablets	55.7	54.7	55.2
Syrups	26.5	25.1	25.8
*Quantity*			
1 tablet/day	41.2	53.7	47.2
*Frequency of Medicine*			
2-3 times per year	33.6	22.7	28.4
*Time of Medicine Intake*			
After meals	42.2	25.0	34.2
At bed times	21.6	27.3	24.3
*Duration*			
Years	31.7	51.7	41.3
*Purchase*			
Medical store	80.2	67.1	73.8
*Addicted to Medicine Used*			
No	86.0	82.3	84.2

While eliciting opinion about self-medication, 29% declared it harmful, 18.5% said consultation is better, while the rest of cases declared it good as it save time and money (15.9%), it works (10.3%), the doctors prescribed the same (10%) and it is good for minor ailments (6.4%).

Side-effects were experienced by 19.3% and 85.4% were benefited. Main source of medicines for males were friends (42.5%) and for females were relatives (30.4%). In this survey 73.8% purchased the medicine from medical stores while 48.5% also recommended those medicines to others [Table-III]. The most common purposes for self-medication were in order of frequency; headache and pain (69.6%) and fever (14.4%) for males, and headache and pain (68.4%) and diarrhea (7.6%) for females see [Table-IV].

**Table-IV:** Purpose of self-medication.

Purpose	Headache and pain	Sore throat/cough	Fever	Skin and hair problems	Diarrhea	Allergy	Weight loss	Energy	Others
Males %	69.6%	10.4%	14.4%	0.8%	1.2%	2.4%	0.8%	–	0.4%
Females %	68.4%	3.6%	4.0%	–	7.6%	4.8%	1.6%	1.2%	8.8%

(Conditions in relation to self-medication in males and females)

## DISCUSSION

This community-based study revealed that the prevalence of self-medication was 84.8% in Karachi as compared to an earlier study published in 1995 ‘where the results were 51%.^11^ The prevalence shown by our study is also high and need to be taken seriously. Highest prevalence rate was found in Gulshan town followed by Defence, North Nazimabad, Orangi town and Malir. The difference was not much among the towns although it was expected that the prevalence will be much lower in well-educated and affluent areas like Defence and Gulshan town mainly due to the reasons that they are aware of self-medication hazards and can afford medical expenses. However, the main reason for doing self-medication came out to be previous experience with similar symptoms.

Although it is true that self-medication can help treat minor ailments that do not require medical consultation and hence reduce the pressure on medical services particularly in the underprivileged countries with limited health care resources^16^ but at the same time practice of self-medication has many adverse effects which may lead to problems like multidrug resistant pathogens^17^, hazards of misdiagnosis^18^, over and under dosing problems^19^, drug interaction^20^, tragedies relating to side effects of drugs^21^ and drug dependence& addiction. 22

A phenomenon is more prevalent in males as compared to females because hospital facilities are available only in morning time and at that time males are at their working places. It was also revealed during the study that some of the participants felt that they indulged in self-medication because doctors advice tests that are time consuming, costly and sometimes lost during procedures.

The source of medicine in males was friends and for females it was relatives. Doctor’s previous prescription and medical stores had also contributed to it. This point should also be taken into account and strategies should be made to prevent the supply of medicine without prescription by pharmacies.

A high percentage of people (52.7% males and 3.9% females) were found continue to recommend medicines to others without realizing that it could be dangerous to others hence the lay person should be educated about the dangers of indiscriminate use of medicines.

It is important to note that antibiotics were used by 1.1% of population without prescription that is comparable to the prevalence rate of 0.1%-21% in Europe^3^ and this is serious because it may lead to antibiotics resistance which need to be highlighted. Physicians should be more judicious in prescribing, and must insist on drugs being supplied by the chemist only on a valid prescription.

A similar study was carried out on Gastro-esophageal reflex in Europe in 2006 and the prevalence of self-medication was found to be 68%.^4^ A study carried on the same topic by Aga Khan Medical students among medical and non-medical students in 2008 showed the prevalence of 76%.^23^

### Limitations of the study

Since this was a community-based study, it was found difficult to communicate with general public mainly due to security issues, people especially in Defense area were reluctant to open their doors, and others who responded some of them were highly none co-operative. In addition, due to lack of finances and time, we restricted our sample size to 500. The field of study can also be expanded in terms of compliance rate.

## CONCLUSION

Self-medication is highly prevalent (84.8%) in Karachi. The main reasons for its use were headache followed by fever. Predominantly painkillers, fever reducers and anti-tussive were used in the form of tablets and syrups by both genders. Self-medications mostly recommended by friends to males and by relatives to females.
